# Environmental variables influence the developmental stages of the citrus leafminer, infestation level and mined leaves physiological response of Kinnow mandarin

**DOI:** 10.1038/s41598-021-87160-8

**Published:** 2021-04-08

**Authors:** Rab Nawaz, Nadeem Akhtar Abbasi, Ishfaq Ahmad Hafiz, Muhammad Faisal Khan, Azeem Khalid

**Affiliations:** 1 Department of Horticulture, Pir Mehr Ali Shah- Arid Agriculture University Rawalpindi, Rawalpindi, Pakistan; 2 Department of Environmental Sciences, Pir Mehr Ali Shah- Arid Agriculture University Rawalpindi, Rawalpindi, Pakistan

**Keywords:** Physiology, Plant sciences

## Abstract

Climate change has not only exacerbated abiotic stress, but has also rendered external conditions more feasible for pests to spread and infest citrus fruit. Citrus leafminer (*Phyllocnistis citrella*) is a potential pest that directly feeds the newly sprouted leaves and twigs of all three spring, summer and autumn flushes. Increasing temperatures in spring and autumn, leafminer accrued more heat units or developmental degree days to accelerate the biological stages of its life-cycle, thereby increasing the pressure of infestation. Present work was conducted at three different environmental conditions in Sargodha, Toba Tek Singh (TTS) and Vehari districts of the Punjab province, Pakistan; all three experimental sites were located in different agro-ecological zones. More infestation was recorded in all three flushes at TTS and Vehari than in Sargodha. Overall, more damage was observed due to higher temperatures in TTS and Vehari than in Sargodha. After May–June heat stress, spontaneous vegetative growth continued from July to November, produced newly spouted tender leaves for feeding the leafminer larvae, and was seen more in TTS and Vehari. Leafminer larva prefers to enter young and tender leaves with a maximum entrance in leaves up to 1 cm^2^ in size while observing no entrance above 3 cm^2^ of leaf size. Physiological response of leaves primarily attributed to chlorophyll and carotenoid contents, both of which were recorded lower in the mined leaves, thereby reducing leaf photosynthetic activity. Similarly, lower levels of polyphenols and antioxidant activity were also recorded in the mined leaves. The on-tree age of mined leaves of three vegetative flushes of Kinnow plant was also less counted than non-mined leaves. Climate change has affected vegetative phenology and become feasible for pests due to extemporaneous leaf growth, particularly leafminer, and eventually causes economic loss by supplying low carbohydrates either to hanging fruits or next-season crops.

## Introduction

Citrus fruit ranks first in the area and production among fruit growing in Pakistan, and the province of Punjab holds a dominant 95 percent share of area under citrus cultivation with the same production level^1^. Among the citrus cultivars in the Punjab plain, Kinnow mandarin grows 91% in three major clusters in the central-north, central and southern regions of the province^2^. The central-north cluster representing Sargodha and the adjacent districts of M.B Din and Chiniot leads the citrus area (60–62%), the central cluster area (8–10%) comprising the districts of TTS, Faisalabad and Jhang, and the southern cluster area (15–18%) including the districts of Sahiwal, Khanewal and Vehari, all three clusters are located in the irrigated canal area^3^.

Approximately 34 insects and mite pests have infested various citrus cultivars^3,4^, including citrus leafminer (CLM) or leafminer (LM) *Phyllocnistis citrella* Stainton (Lepidoptera: Gracillariidae), which has emerged as a direct leaf damaging citrus pest and associated ornamental plants^5^. It is a destructive pest^6^, with 16 generations overlapping in one year^7^ and likely to attack nurseries, young plantations and tender leaves^8^. The leafminer larva makes serpentine mines in the tender leaf epidermis, and during the pupation period fold leaves to avoid external threats^9^. Average infestation of leafminer ranges from 17 to 57%^10^ and mostly spring and summer flushes are more infested than autumn. Fruit development is dependent on spring flush photoassimilates, while summer flush encourages citrus canker entry^11,12^. Leafminer infestation directly reduced yield and caused stunted plant growth and an early drop of infested leaves on all three vegetative flushes^13^. Henceforth, mined leaves have imperfect physiological response^14^ with low photosynthetic activity^15,16^ and also possess less polyphenols and antioxidant activity^17^ by directly reducing on-tree age of infested leaf.

Changes in climatic factors not only caused abiotic stress on plants, but also increased biotic incidence^18^. In the climate change scenario, temperature rise^19^ has become suitable for invasive species^20^ and optimized conditions for insects migration^21,22^. The distribution of the insect population depends on temperature^23^ and the temperature increase in early spring optimized the oviposition of the leafminer and the highest oviposition rate was recorded at 3 °C and the lowest at 20 °C^24^ and the oviposition period was shortened during high temperatures. The life cycle of the leafminer lasted 13 to 45 days depending on the temperature^25,26^, the egg, larval and pupal periods squeezed with rising temperature^8^ and shortened during the summer^27^. Temperature, average relative humidity, and sunshine period are used to count agrometeorological indices of a specific area^28–31^ with year-round fluctuating patterns in different seasons^32^ by counting more in warm regions^33,34^. Insect-pest developmental stages are affected by agrometeorological indices such as developmental degree days (DDs)^35^ and photoperiod^36,37^, while climate variables also affect the developmental stages of leafminer^38^. Meteorological indices determine the duration of the life cycle^39,40^ and the number of generations of leafminer per year^41,42^ by indicating population pressure on citrus in a particular area^40,43^ and also availability of new flush^25,44^.

Climate change in Pakistan has elevated temperature, extended summer spell, squeezed autumn and spring seasons, and caused erratic weather in the winter months^2^. On the one hand, insect-pests accelerated the life cycle by the accumulation of more heat units/degree days (DDs) and, on the other hand, caused serious environmental stress to the plant. Prior to this, research has been carried out on citrus pests pertaining to Integrated Pest Management (IPM), but the impact of climate change on citrus fruit remains to be explored. In this context, the present study was conducted in the main citrus groves/clusters of the Punjab province in three districts, namely Sargodha, Toba Tek Singh (TTS) and Vehari, all of which have different levels of meteorological/agrometeorological indices affecting the developmental stages of the leafminer. Similarly, an evaluation of leafminer damaged percentage on citrus (Kinnow mandarin) three vegetative flushes, leaf size damaged percentage, physiological response, chlorophylls, carotenoids, polyphenols contents and antioxidant activity of non-mined and mined leaves under varying environmental conditions was conducted.

## Materials and methods

The present study was conducted in three major citrus growing regions of the province of Punjab, Pakistan, during the 2017–18 and 2018–19 seasons.

### Weather data of three locations

Weather data from the respective experimental sites were taken from the Pakistan Meteorological Department and the average data for two years (2017 and 2018) is shown in Table 1.

**Table 1:** Weather data of three districts.

	Weather data (2017 and 2018) of three research sites											
	Average temperature (°C)			Average relative humidity (%)			Rainfall (mm)			Actual sunshine (h)		
	SGD	TTS	VEH	SGD	TTS	VEH	SGD	TTS	VEH	SGD	TTS	VEH
Jan	12.17	12.76	14.13	80.05	81.05	73.68	18.84	5.41	7.50	114.60	145.70	155.25
Feb	16.34	16.80	17.26	65.93	63.48	61.65	14.02	6.41	5.50	188.80	190.95	198.75
Mar	21.62	22.13	24.94	63.68	65.32	57.98	26.30	13.96	3.50	222.15	219.65	234.31
Apr	27.56	28.73	31.24	53.67	51.27	41.03	73.59	44.73	1.00	250.70	237.15	250.80
May	31.56	32.39	35.83	49.35	46.98	32.85	39.03	21.37	2.00	256.05	230.65	255.31
Jun	32.59	32.80	35.78	59.00	62.85	40.70	58.84	48.96	38.50	215.25	189.05	255.25
Jul	31.57	32.96	33.20	72.47	71.37	55.22	167.17	102.46	81.00	211.75	218.80	230.45
Aug	31.98	32.62	33.03	70.87	68.76	55.73	52.53	79.06	1.00	214.95	208.45	238.75
Sep	29.66	30.78	33.88	70.63	67.13	51.87	49.02	20.96	0.00	230.95	222.55	233.90
Oct	25.56	26.93	29.65	63.76	64.60	61.00	3.01	0.01	0.00	225.10	219.55	229.40
Nov	18.03	18.84	19.78	80.02	82.40	64.42	1.53	2.51	0.00	187.40	131.20	138.75
Dec	13.43	14.56	16.65	72.23	72.94	71.60	7.52	4.06	4.00	199.95	181.45	193.80
Avg	24.34	25.19	27.11	66.80	66.51	55.64	42.61	29.15	12.00	209.80	199.60	217.89

### Selection of orchards for data collection

The selected orchards were located at Sargodha (32.0837° N, 72.6719° E and altitude 189 m), Toba Tek Singh (30.9727° N, 72.4850° E and altitude 161 m) and Vehari (30.0452° N, 72.3489° E and altitude 140 m) in Pakistan. Plants of identical features like age (12–15 years), healthy, vigorous with planting density (250–260 plants/ha) in block form having 2 hectares area were selected from three sites. Total 36 branches/twigs of lead pencil size were marked at different canopy positions of the plant^44^. Tagged branches represented three canopy positions (lower, middle and upper) from plant four directions and at each canopy position (inner, middle and outer) sides were used. Uniform cultural practices were followed in experimental sites. Data were recorded on a monthly basis during the growing season 2017–2018 and 2018–2019^31^.

### Agrometeorological/Thermal indices calculation

#### Leaf miner threshold temperature and thermal constant

Threshold temperature of the leaf miner developmental stages (10.57, 7.31 and 7.42 °C) and thermal constant/developmental degree days (50.76, 109.89 and 136.98 DDs) of eggs, larvae and pupae, respectively, were used in the calculation of agrometeorological/thermal indices^42^.

#### I-Developmental degree days (DDs)

Threshold temperature of each developmental stage of leafminer (egg to pupa) was subtracted on a monthly basis from the mean daily temperature and expressed as °C day^28,30,45^.

$${\text{DDs}} = \frac{{{\text{T}_{\max}} + {\text{T}_{\min}}}}{2} - {\text{T threshold of individual stage }}$$

#### II-Hydrothermal units (HYTUs)

Leafminer's individual developmental stages DDs were multiplied with each month's average relative humidity (RHa) for the calculation of HYTUs and expressed as °C day%^30,46,47^.

$${\text{HYTUs}} = {\text{ DDs }} \times {\text{ average relative humidity }}\left( {{\text{RHa}}} \right)$$

#### III-Photothermal index (PTI)

Individual stage developmental degree days (DDs) of leafminers were divided on the respective month time taken in days to complete the cycle of different phases and expressed in °C^30,48,49^.

$$\begin{aligned} & {\text{Monthly basis days taken by individaul stage }} \\ & \quad = \frac{{\text{Montly accumulated DDs}}}{{{\text{Individual stage DDs }}\left( {\text{threshold constant}} \right)}} \\ & \quad \times {\text{ No}}.{\text{ of days in respective month }} \\ & {\text{PTI}} = \frac{{\text{Monthly accumulated DDs}}}{{\text{Monthly basis days taken by individual satge}}} \\ \end{aligned}$$

#### IV-Helio thermal unit (HTU)

Actual bright sunshine hours were multiplied by DDs and value articulated in °C day hours^30,44^.

$${\text{HTU}}\left( {^\circ {\text{C day}}} \right) = {\text{DDs}}\, \times \,{\text{actual bright sunshine hours}}$$

### Different level DDs counted in climate change

Historical temperature data (1983–2016) of experimental sites (available on Agroclimatology Parameters on NASA website) was used to count developmental degree days (DDs) of leafminer by keeping developmental stage (egg to pupa) threshold temperature (8.43 °C). Average temperature based DDs were calculated for the study period (2017 and 2018) and compared to previous data (1983–2000) and (2001–2016) to obtain monthly basis differences in the availability of DDs to leafminer. A gradual mean daily temperature has risen due to global warming by endowing more DDs to leafminer. Additional accumulation of DDs indicates rapid wrapping of leafminer developmental stages, which were counted more under warm Vehari and TTS conditions indicating increased population pressure. Data are shown in Table 2.

**Table 2:** Monthly basis DDs computation of three sites.

	Monthly available developmental degree days (DDs) of leafminer (egg to pupation stages)								
	Sargodha			T.T Singh			Vehari		
	Average (2017–2018) (°C day)	Average (2001–2016) (°C day)	Average (1983–2000) (°C day)	Average (2017–2018) (°C day)	Average (2001–2016) (°C day)	Average (1983–2000) (°C day)	Average (2017–2018) (°C day)	Average (2001–2016) (°C day)	Average (1983–2000) (°C day)
Jan	116.07	110.65	80.84	134.17	121.56	100.29	176.67	190.68	137.53
Feb	221.46	194.52	145.86	234.29	228.61	167.12	247.21	249.01	169.09
Mar	408.95	399.63	327.58	424.82	441.03	361.41	511.67	473.50	384.35
Apr	571.25	570.22	501.70	608.98	615.79	530.06	684.35	643.19	599.87
May	716.92	710.3	673.29	742.72	735.65	698.14	849.42	830.58	747.38
Jun	721.60	765.35	750.78	731.08	765.36	758.08	820.35	864.13	787.35
Jul	717.85	730.25	720.59	760.32	748.35	735.5	768.67	770.35	758.81
Aug	730.05	757.18	620.08	749.97	758.65	691.73	762.67	775.81	714.95
Sep	636.83	659.31	592.90	670.43	689.31	610.6	763.35	691.21	676.66
Oct	531.05	563.6	476.23	573.47	545.60	500.79	657.67	603.33	548.10
Nov	287.98	316.5	306.33	312.28	325.25	317.04	340.60	408.67	348.30
Dec	154.95	165.35	147.05	189.93	175.36	168.33	254.67	259.28	196.31
Total	5814.96	5942.86	5343.23	6132.46	6150.52	5639.09	6837.30	6759.74	6068.70

### Quantification of flushes and leafminer mined leaves

The number of leaves in each flush was calculated from the tagged branches and the percentage of flush was quantified by adding three flush leaves^50^. Similarly, in each flush, the mined leaves were also counted, and their percentage was calculated from the total number of mined leaves of each flush.

$${\text{Flush leaves \% }} = \frac{{{\text{Total No}}.{\text{ of leaves of three flushes }}\left( {{\text{spring}} + {\text{summer}} + {\text{autumn}}} \right)}}{{{\text{Respective flush No}}.{\text{ of leaves}}}} \times 100$$

### Monthly sprouting and leafminer larva entrance

Non-mined and mined leaves were calculated on a monthly basis from tagged branches, and the newly sprouted and leafminer larva entry leaves were also calculated.

$$\begin{aligned} & {\text{Monthly newly sporuted leaves}} \%  \\ & = \frac{{{\text{Total No}}.{\text{ of leaves sprouted in each month in year}}}}{{{\text{Respective month No}}.{\text{ of sprouted leaves}}}} \times 100 \\ \end{aligned}$$

### Leaf size entrance by larva after egg-hatching

Leaf area meter (Model: Li-3100C Area Meter, Li-COR, Biosciences) was used to measure Kinnow leaves by size of leafminer infestation/mining. Damaged/mined leaves (1 cm) of mining length (1st instar larva) were detached from the plant and the leaf area was measured. 1 cm of mining leaves were detached in order to count the size of the leaf, which 1st instar larvae prefer to enter for feeding just after the egg-hatching. Mining length (1 cm) represents first instar larva which has taken 4–5 days after egg hatching^51^.

### Chlorophylls and carotenoids measurement

The method followed by^52,53^ was used to measure the chlorophyll and carotenoid levels of the mined and non-mined leaves, using the equations described by^54^.

$$\begin{aligned} {\text{Total Chlorophyll}} & = {2}0.{\text{2 A645}} + {8}.0{\text{2 A663}} \\ {\text{Chlorophyll a }}\left( {{\text{Ca}}} \right) & = {11}.{\text{75 A663 }} - { 2}.{35}0{\text{ A645}} \\ {\text{Chlorophyll b }}\left( {{\text{Cb}}} \right) & = {18}.{\text{61 A645 }} - { 3}.{96}0{\text{ A663}} \\ {\text{Total carotenoids}} & = {1}000{\text{ A47}}0 \, - { 2}.{27}0{\text{ Ca }} - { 81}.{\text{4 Cb}}/{227} \\ \end{aligned}$$

### Polyphenols and anti-oxidant measurement

Antioxidant activity (DPPH inhibition %) and total phenolic contents were measured using the^55^ method, while total flavonoids and flavonol contents were also determined using the^36,56^ procedure. The standard gallic acid curve used by^57^ to calculate the total phenolic contents and the standard quercetine curve of^58^ was used to estimate the total flavonoids and flavonol contents.

### Physiological response of non-mined and mined leave

Portable Photosynthesis System (Model: PP-Systems, CIRAS-3, Amesbury, U.S.A) was used to measure physiological response of non-mined and mined leaves. Net assimilation rate, stomatal conductance, sub-stomatal conductance/intercellular CO_2_ concentration, transpiration rate and water use efficiency parameters were measured. Ambient sunlight and cuvette temperature with a standard reference of CO_2_ (390 μmol mol^−1^) have been fixed (manual of the instrument available at www.ppsystems.com) for measuring data of different mining lengths and intact leaves. The selected leaves were removed after taking photosynthetic activities to measure mining length. Mined area of the leaf was removed to measure percentage of damaged leaf. Data recorded on the PP-System/Infrared Gas Analyzer (IRGA) of different leaves with mining damage ranged from 10 to 60 per cent compared to non-mined leaves. Matured two-month-old (mined and non-mined) leaves were used to estimate physiological response. Similarly, different age in months^1–8^ of both non-mined and mined leaves physiological response was also checked through IRGA. Leaf cuvette dimension (18 × 25 mm) was set to cover an area of 4.5 cm^2^. Whole chamber was covered with leaf by inserting middle side of non-mined and infested portion of mined leaves to attain accuracy. Similarly identical features leaves free from insect-pests except LM of both non-mined and mined were used.

### Leaf sclerophylly

Leaf sclerophylly parameters like leaf area, fresh weight (FW), dry weight (DW), specific leaf area (SLA = LA/DW: in cm^2^ g^−1^ DW), specific leaf weight (SLW = DW/LA: in g cm^2^ LA), density of foliar tissue (D = DW/FW X 1000: in g kg^−1^) and succulence [S = (FW-DW)/LA: in mg H2O cm^−2^] were used in all three flushes having (10–60 days) leaf age^59^.

### Leaf age calculation of non-mined and mined leaves of three flushes

At different times of both mined and non-mined leaves of each flush leaf age was counted from labeled branches from the sprouting date to the leaf-shedding.

### Statistical analysis

The research was designed in randomized complete block design using factorial analysis on the Statistix 8.1 software. Analysis of variance of the individual parameter was performed using LSD while keeping *P* value at *P* ≤ 0.05. In tables data are shown in means ± standard deviation (M ± SD) and in figures means ± standard error (M ± SE). In each replicate, three plants of uniform features like age, health and canopy volume were used by tagging 36 branches of uniform size at analogous canopy positions of individual tree in all three experimental sites to attain uniformity.

## Results

Data analysis regarding leafminer damage and physiological response of mined leaves reflected significant influence under varying environmental conditions, which were explained and discussed under.

### Agrometeorological/Thermal indices

Agrometeorological/thermal indices availability in different developmental stages of leafminer is presented in Tables 3, 4, 5, 6 and 7. Mean daily temperature was observed higher in warm districts of Vehari, followed by TTS and lower in Sargodha, and the availability of leafminer developmental degree days (DDs) was computed accordingly. Maximum DDs (884.14 °C day) were calculated during May at Vehari in the larva stage and minimum (49.73 °C day) at Sargodha in the egg-hatching stage during the month of January. More time was taken at Sargodha in the cool month of January (31.64 days) and less time in the warm month of May at Vehari (2.01 days) in the egg-hatching stage. Leafminer egg-hatching stage took more number of days in cool months at Sargodha and less was counted at Vehari in summer months. More photothermal indexes were recorded in May at Vehari (389.7 °C) and less in Sargodha (1.57 °C) during January at the egg-hatching stage. PTI level was increased in warm months and more availability was observed during the egg-hatching stage. Maximum helio thermal unit was available at Vehari (225,729.78 °C day hours) during the developmental stage of the larva and minimum was computed at Sargodha (5699.06 °C day hours) during the egg-hatching stage in January. Hydrothermal units (HYTUs) were more available in July at TTS (56,340.74 °C day percent) at the developmental stage of larvae and less in January at Sargodha (3980.89 °C day percent) at the egg-hating stage. Significant differences in agrometeorological indices were observed on a monthly basis in the developmental stages of leafminer and districts.

**Table 3:** Leafminer developmental degree days (DDs).

	Leafminer different stages monthly developmental degree days(DDs)								
	Sargodha			T.T Singh			Vehari		
	Egg (°C day)	Larva (°C day)	Pupa (°C day)	Egg (°C day)	Larva (°C day)	Pupa (°C day)	Egg (°C day)	Larva (°C day)	Pupa (°C day)
Jan	49.73	150.79	147.38	67.83	168.89	165.48	110.33	211.39	207.98
Feb	161.54	252.82	249.74	174.365	265.645	262.565	187.29	278.57	275.49
Mar	342.605	443.665	440.255	358.48	459.54	456.13	445.33	546.39	542.98
Apr	507.025	604.825	601.525	544.775	642.575	639.275	620.15	717.95	714.65
May	650.58	751.64	748.23	676.38	777.44	774.03	783.08	884.14	880.73
Jun	657.4	755.2	751.9	666.875	764.675	761.375	756.15	853.95	850.65
Jul	663.705	764.765	761.355	683.63	784.69	781.28	696.33	797.39	793.98
Aug	572.625	670.425	667.125	606.225	704.025	700.725	699.15	796.95	793.65
Sep	572.625	670.425	667.125	606.225	704.025	700.725	699.15	796.95	793.65
Oct	464.705	565.765	562.355	507.13	608.19	604.78	591.33	692.39	688.98
Nov	223.775	321.575	318.275	248.075	345.875	342.575	276.4	374.2	370.9
Dec	88.605	189.665	186.255	123.63	224.69	221.28	188.33	289.39	285.98
Total	4954.92	6141.56	6101.52	5263.62	6450.26	6410.22	6053.02	7239.66	7199.62

**Table 4:** Leafminer developmental stages time taken in days.

	Monthly number of days taken in leafminer different developmental stages								
	Sargodha			T.T Singh			Vehari		
	Egg (days)	Larva (days)	Pupa (days)	Egg (days)	Larva (days)	Pupa (days)	Egg (days)	Larva (days)	Pupa (days)
Jan	31.64	22.59	28.81	23.20	20.17	25.66	14.26	16.12	20.42
Feb	8.80	12.17	15.36	8.15	11.58	14.61	7.59	11.05	13.92
Mar	4.59	7.68	9.65	4.39	7.41	9.31	3.53	6.23	7.82
Apr	3.00	5.45	6.83	2.80	5.13	6.43	2.46	4.59	5.75
May	2.42	4.53	5.68	2.33	4.38	5.49	2.01	3.85	4.82
Jun	2.32	4.37	5.47	2.28	4.31	5.40	2.01	3.86	4.83
Jul	2.37	4.45	5.58	2.30	4.34	5.44	2.26	4.27	5.35
Aug	2.75	5.08	6.37	2.60	4.84	6.06	2.25	4.27	5.35
Sep	2.66	4.92	6.16	2.51	4.68	5.86	2.18	4.14	5.18
Oct	3.39	6.02	7.55	3.10	5.60	7.02	2.66	4.92	6.16
Nov	6.81	10.25	12.91	6.14	9.53	12.00	5.51	8.81	11.08
Dec	17.76	17.96	22.80	12.73	15.16	19.19	8.36	11.77	14.85
Avg	7.38	8.79	11.10	6.04	8.10	10.20	4.59	6.99	8.79

**Table 5:** Leafminer developmental stages photothermal index.

	Leafminer different developmental stages monthly photothermal index (PTI)								
	Sargodha			T.T Singh			Vehari		
	Egg (°C)	Larva (°C)	Pupa (°C)	Egg (°C)	Larva (°C)	Pupa (°C)	Egg (°C)	Larva (°C)	Pupa (°C)
Jan	1.57	6.67	5.12	2.92	8.37	6.45	7.74	13.12	10.19
Feb	18.36	20.77	16.26	21.39	22.93	17.97	24.68	25.22	19.79
Mar	74.59	57.78	45.64	81.67	61.99	49.00	126.03	87.64	69.43
Apr	168.82	110.96	88.05	194.89	125.25	99.45	252.55	156.35	124.28
May	268.98	165.84	131.84	290.74	177.42	141.09	389.70	229.47	182.67
Jun	283.80	173.00	137.58	292.04	177.37	141.06	375.47	221.20	176.09
Jul	279.94	171.69	136.51	297.00	180.75	143.75	308.14	186.65	148.46
Aug	208.38	131.94	104.81	233.55	145.50	115.63	310.64	186.44	148.33
Sep	215.33	136.34	108.30	241.34	150.35	119.49	320.99	192.66	153.28
Oct	137.24	93.96	74.47	163.44	108.58	86.13	222.22	140.73	111.79
Nov	32.88	31.37	24.65	40.41	36.29	28.56	50.17	42.47	33.48
Dec	4.99	10.56	8.17	9.71	14.82	11.53	22.54	24.58	19.26
Avg	141.24	92.57	73.45	155.76	100.80	80.01	200.91	125.54	99.75

**Table 6:** Leafminer helio thermal units (HTU).

	Leafminer different stages monthly helio thermal unit (HTU)								
	Sargodha			T.T Singh			Vehari		
	Egg (°C day h)	Larva (°C day h)	Pupa (°C day h)	Egg (°C day h)	Larva (°C day h)	Pupa (°C day h)	Egg (°C day h)	Larva (°C day h)	Pupa (°C day h)
Jan	5699.06	17,280.53	16,889.75	9882.83	24,607.27	24,110.44	17,128.73	32,818.30	32,288.90
Feb	30,498.75	47,732.42	47,150.91	33,295.00	50,724.91	50,136.79	37,223.89	55,365.79	54,753.64
Mar	76,109.70	98,560.18	97,802.65	78,740.13	100,937.96	100,188.95	104,345.27	128,024.64	127,225.64
Apr	127,111.17	151,629.63	150,802.32	129,193.39	152,386.66	151,604.07	155,533.62	180,061.86	179,234.22
May	166,581.01	192,457.42	191,584.29	156,007.05	179,316.54	178,530.02	199,928.15	225,729.78	224,859.18
Jun	141,505.35	162,556.80	161,846.48	126,072.72	144,561.81	143,937.94	193,007.29	217,970.74	217,128.41
Jul	140,539.53	161,938.99	161,216.92	149,578.24	171,690.17	170,944.06	160,469.25	183,758.53	182,972.69
Aug	123,085.74	144,107.85	143,398.52	126,367.60	146,754.01	146,066.13	166,922.06	190,271.81	189,483.94
Sep	132,247.74	154,834.65	154,072.52	134,915.37	156,680.76	155,946.35	163,531.19	186,406.61	185,634.74
Oct	104,605.10	127,353.70	126,586.11	111,340.39	133,528.11	132,779.45	135,651.10	158,834.27	158,052.01
Nov	41,935.44	60,263.16	59,644.74	32,547.44	45,378.80	44,945.84	38,350.50	51,920.25	51,462.38
Dec	17,716.57	37,923.52	37,241.69	22,432.66	40,770.00	40,151.26	36,498.35	56,083.78	55,422.92
Total	1,107,635.16	1,356,638.85	1,348,236.88	1,110,372.83	1,347,337.01	1,339,341.29	1,408,589.41	1,667,246.35	1,658,518.66

**Table 7:** Leafminer hydrothermal units (HYTUs).

	Leafminer different stages monthly hydrothermal units (HYTUs)								
	Sargodha			T.T Singh			Vehari		
	Egg (°C day %)	Larva (°C day %)	Pupa (°C day %)	Egg (°C day %)	Larva (°C day %)	Pupa (°C day %)	Egg (°C day %)	Larva (°C day %)	Pupa (°C day %)
Jan	3980.89	12,070.74	11,797.77	5226.30	13,012.97	12,750.23	8144.01	15,603.75	15,352.04
Feb	10,647.91	16,664.63	16,461.61	11,294.49	17,207.15	17,007.65	11,547.36	17,175.23	16,985.34
Mar	21,820.51	28,257.02	28,039.84	22,799.33	29,226.74	29,009.87	25,301.42	31,043.15	30,849.41
Apr	27,219.64	32,470.03	32,292.87	28,055.91	33,092.61	32,922.66	25,441.65	29,453.90	29,318.52
May	32,109.38	37,097.19	36,928.89	31,299.48	35,976.04	35,818.24	29,071.85	32,823.70	32,697.10
Jun	38,786.60	44,556.80	44,362.10	40,929.45	46,931.93	46,729.39	36,949.27	41,728.27	41,567.01
Jul	48,092.06	55,414.87	55,167.78	49,084.63	56,340.74	56,095.90	37,016.90	42,389.25	42,207.98
Aug	40,590.52	47,523.08	47,289.16	41,950.77	48,718.53	48,490.17	39,491.49	45,015.72	44,829.32
Sep	40,450.23	47,358.82	47,125.71	41,011.12	47,627.29	47,404.05	37,488.42	42,732.46	42,555.51
Oct	29,627.27	36,070.35	35,852.94	33,331.12	39,973.29	39,749.17	35,671.98	41,768.43	41,562.72
Nov	17,904.24	25,729.22	25,465.18	19,095.57	26,623.73	26,369.71	18,098.67	24,502.62	24,286.53
Dec	6398.61	13,696.66	13,450.40	8932.27	16,233.85	15,987.48	14,840.40	22,803.93	22,535.22
Total	317,627.85	396,909.41	394,234.26	333,010.46	410,964.88	408,334.52	319,063.44	387,040.40	384,746.70

### Kinnow flushes and leafminer damage

**Figure 1:**
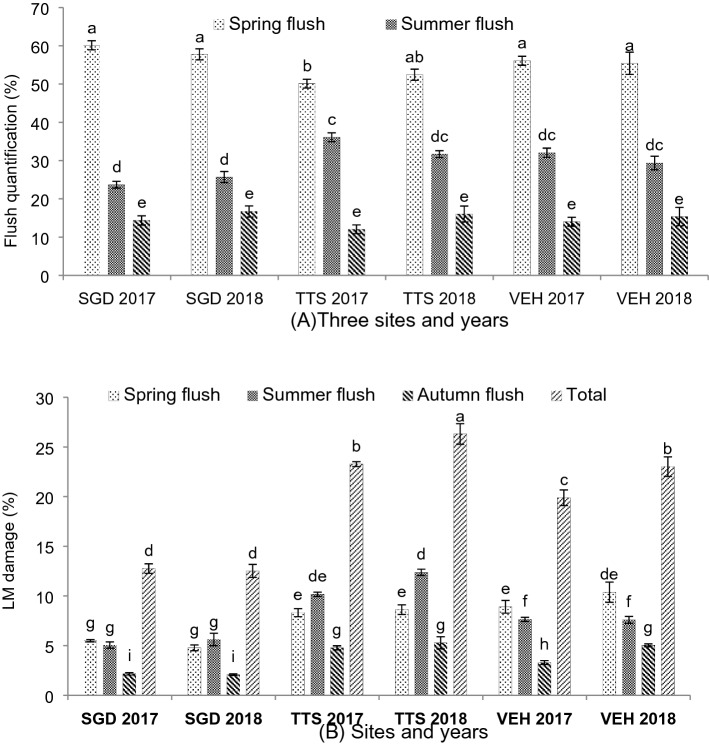
(**A**) Quantification of three vegetative flushes (**B**) Leafminer damage of vegetative flushes (leaves percentage). Bars sharing dissimilar letters are significantly differed according to LSD test (*P* ≤ 0.05).

Estimation of vegetative flush and leafminer damage on each flush was measured in three different ecological zones of Punjab, with a substantial statistical difference as shown in Fig. 1. Spring flush was recorded as maximum in Sargodha (60%) and minimum at TTS (52%) during 2017 season. Summer flush was quantified higher in TTS (36%) and lower at Sargodha (23.67%) in season 2017. Autumn flush was counted more at Sargodha (16.67%) in growing year 2018 and less in TTS (12%) during 2017. Mined leaves were seen in spring flush as higher in Vehari (10.39%) and lower at Sargodha (4.79%) in 2018 season. In summer flush, maximum mined leaves were recorded at TTS (12.39%) in season 2018 and minimum in Sargodha (5.07%) during 2017. However, in autumn flush, mined leaves higher counting was recorded at TTS (5.29%) and lower in Sargodha (2.11%) during 2018 season. Overall mined leaves were counted as maximum at TTS (26.31%) and minimum in Sargodha (12.52%) during 2018. It was statistically insignificant differences in the vegetative flush of all three districts during 2017 and 2018. However, due to different levels of pest pressure, a marginal difference in leafminer infestation was observed during both years.

### Monthly sprouting and leafminer larva entrance

**Figure 2:**
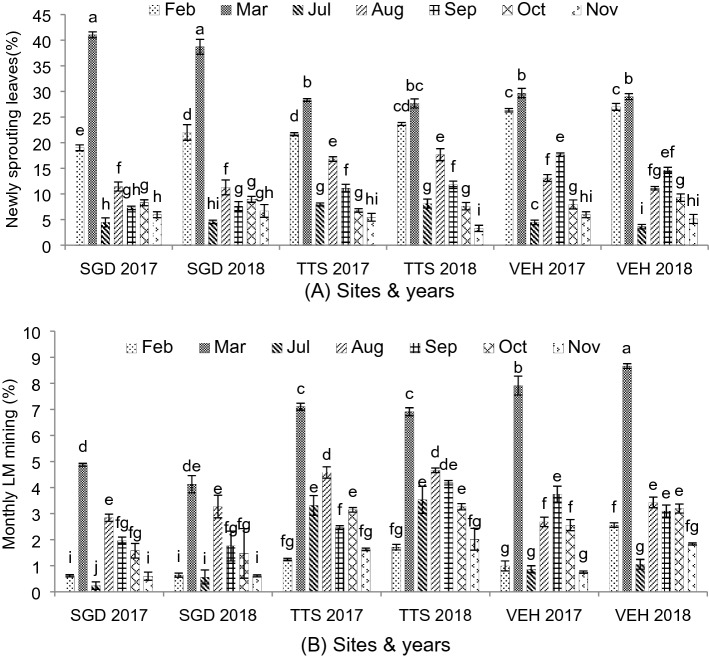
(**A**) Monthly newly sprouted leaves (**B**) Monthly leafminer mining on newly sprouted leaves. Bars sharing dissimilar letters are significantly differed according to LSD test (*P* ≤ 0.05).

The perusal of data on monthly new sprouting leaves and damaged leaves from leafminer was shown in Fig. 2A,B, which showed significant impacts of different climatic conditions. Maximum newly sprouted leaves were recorded at Sargodha (41%) during March 2017 and minimum in TTS (3.33%) in the month of November 2018. In all three experimental sites, higher count of newly sprouting leaves were seen in March and lower during July and November. Similarly higher LM infestation was seen in March and lower in February and November in all three sites. However, maximum mined leaves were counted at Vehari (8.66%) in March 2018 and lower in Sargodha (0.25%) in the month of July 2017. In all three districts, no new sprouting was seen in January, April, May, June and December and henceforth, LM infestation was not recorded as such in these months, as larva just after egg-hatching prefer to feed or make zigzag entry in newly emerging leaves.

### Leaf size entrance by larva after egg-hatching

**Figure 3:**
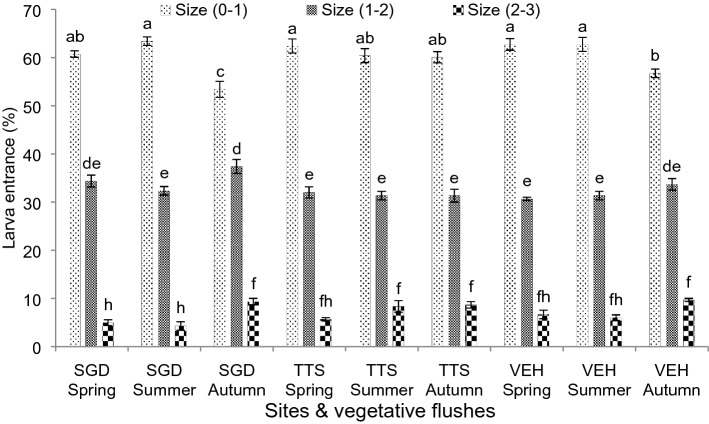
Larva entrance after egg-hatching of leaf size. Bars sharing dissimilar letters are significantly differed according to LSD test (*P* ≤ 0.05).Leaf size in cm^2^.

Leaf size mined by leaf miner was presented in Fig. 3. Maximum larva entry/mining was recorded in leaf size (0–1 cm^2^), followed by leaf size (1–2 cm^2^) and least in leaf size (2–3 cm^2^) at all three districts. In leaf size (0-1cm^2^), maximum larva entry was found (63.33%) in spring flush and minimum (53.33%) in autumn flush at Sargodha. In leaf size (1-2cm^2^), higher larva mining was recorded at Sargodha (37.33%) in autumn flush and lower at Vehari (30.67%) in spring flush. However, least mining was recorded in leaf size (2-3cm^2^) by recording more in autumn flush at Vehari (9.67%) and less in summer flush at Sargodha (4.33%). Leaf size above 3 cm^2^ was shown to be tolerant of newly hatched larva making mine or leaf entry in all three locations. In all three districts and flushes a significant differences were observed with respect to leaf size in the larva making mine or entry.

### Leaf sclerophylly for leafminer larva feeding after entrance

Leaf sclerophylly of three flushes are presented in Tables 8, 9 and 10. In spring flush leaf area (2.66, 4.93, 6.24, 8.03, 9.67 and 11.86 cm^2^) and leaf succulence (0.0205, 0.0235, 0.0219, 0.0209, 0.0193 and 0.0188 mg H_2_O cm^−2^) were recorded at 10th, 20th, 30th, 40th, 50th and 60th days of leaf age, respectively. Similarly, leaf area (2.25, 3.75, 4.73, 6.14, 7.95 and 9.36 cm^2^) and leaf succulence (0.0206, 0.0221, 0.0227, 0.0203, 0.0197 and 0.0185 mg H_2_O cm^−2^) were measured in summer flush at 10th, 20th, 30th, 40th, 50th and 60th days of leaf age, respectively. Whereas, leaf area was estimated (2.03, 2.65, 4.5, 6.19, 7.46 and 8.89 cm^2^) and leaf succulence (0.025, 0.027, 0.026, 0.026, 0.025 and 0.025 mg H_2_O cm^−2^) were recorded in autumn flush at 10th, 20th, 30th, 40th, 50th and 60th days of leaf age, respectively. Both leaf area and succulence decide larva feeding/mining in a leaf while the rest of leaf sclerophylly parameters depend on leaf size and weight, which indirectly indicates the susceptible leaves.

**Table 8:** Leaf sclerophylly parameters of spring flush.

	Spring flush leaf sclerophylly at different level of leaf age					
	10th days	20th days	30th days	40th days	50th days	60th days
Leaf area (cm^2^)	2.66 ± 0.52f.	4.93 ± 0.08e	6.24 ± 0.59d	8.03 ± 0.46c	9.67 ± 0.75b	11.86 ± 1.25a
Leaf fresh weight (g)	0.065 ± 0.012e	0.13 ± 0.005d	0.161 ± 0.032c	0.194 ± 0.015b	0.214 ± 0.081b	0.254 ± 0.038a
Leaf dry weight (g)	0.012 ± 0.002c	0.014 ± 0.003c	0.023 ± 0.002b	0.026 ± 0.001ab	0.028 ± 0.009a	0.03 ± 0.002a
Specific leaf area (cm^2^ g^−1^)	231.87 ± 17.59f.	346.98 ± 6.14b	275.19 ± 25.11d	303.24 ± 11.31c	347.61 ± 22.26b	392.48 ± 18.96a
Specific leaf weight (g cm^2^)	0.004 ± 0.0006a	0.003 ± 0.00047a	0.004 ± 0.0006a	0.0033 ± 0.0007a	0.0029 ± 0.0004ab	0.0026 ± 0.0008b
Density of foliar tissue (g kg^−1^)	162.61 ± 12.58a	149.31 ± 11.58b	146.59 ± 21.4b	136.77 ± 9.77bc	130.88 ± 11.44c	120.23 ± 13.72d
Succulence (mg H_2_O cm^−2^)	0.0245 ± 0.003a	0.0235 ± 0.007a	0.0219 ± 0.003b	0.0209 ± 0.008bc	0.0193 ± 0.008c	0.0188 ± 0.005c

**Table 9:** Leaf sclerophylly parameters of summer flush.

	Summer flush leaf sclerophylly at different level of leaf age					
	10th days	20th days	30th days	40th days	50th days	60th days
Leaf area (cm^2^)	2.25 ± 0.2f.	3.75 ± 0.29e	4.73 ± 0.34d	6.14 ± 0.51c	7.95 ± 0.37b	9.36 ± 0.42a
Leaf fresh weight (g)	0.070 ± 0.009e	0.12 ± 0.008d	0.155 ± 0.007c	0.188 ± 0.021b	0.212 ± 0.022ab	0.232 ± 0.025a
Leaf dry weight (g)	0.02 ± 0.003d	0.038 ± 0.005c	0.048 ± 0.006bc	0.063 ± 0.0074a	0.055 ± 0.0047b	0.06 ± 0.0057a
Specific leaf area (cm^2^ g^−1^)	98.1 ± 6.24c	96.6 ± 5.44c	99.11 ± 6.35c	98.63 ± 7.24c	144.17 ± 11.54b	158.1 ± 15.87a
Specific leaf weight (g cm^2^)	0.01 ± 0.0006a	0.001 ± 0.0005a	0.01 ± 0.0006a	0.01 ± 0.0006a	0.007 ± 0.0005b	0.0065 ± 0.008b
Density of foliar tissue (g kg^−1^)	333.54 ± 14.56a	317.18 ± 16.65b	311.34 ± 18.55b	336.06 ± 13.76a	262.52 ± 16.73c	259.2 ± 14.92c
Succulence (mg H_2_O cm^−2^)	0.0206 ± 0.0025b	0.0221 ± 0.0016a	0.0227 ± 0.001a	0.0203 ± 0.0014b	0.0197 ± 0.001bc	0.0185 ± 0.0025c

**Table 10:** Leaf sclerophylly parameters of autumn flush.

	Autumn flush leaf sclerophylly at different level of leaf age					
	10th days	20th days	30th days	40th days	50th days	60th days
Leaf area (cm^2^)	2.03 ± 0.21f.	2.65 ± 0.09e	4.5 ± 0.21d	6.19 ± 0.27c	7.46 ± 0.32b	8.89 ± 0.26a
Leaf fresh weight (g)	0.061 ± 0.01f.	0.086 ± 0.012e	0.14 ± 0.007d	0.188 ± 0.01c	0.222 ± 0.22b	0.262 ± 0.01a
Leaf dry weight (g)	0.011 ± 0.004d	0.013 ± 0.0075d	0.024 ± 0.0047c	0.03 ± 0.003b	0.035 ± 0.009ab	0.039 ± 0.005a
Specific leaf area (cm^2^ g^−1^)	193.04 ± 7.24d	201.62 ± 12.14bc	192.56 ± 13.69c	207.34 ± 18.67b	214.49 ± 18.42ab	229.9 ± 20.67a
Specific leaf weight (g cm^−2^)	0.0052 ± 0.0005a	0.004 ± 0.0004b	0.005 ± 0.00045a	0.005 ± 0.0004a	0.0047 ± 0.0006ab	0.0044 ± 0.0006b
Density of foliar tissue (g kg^−1^)	172.05 ± 14.56a	153.62 ± 12.85c	165.72 ± 11.56ab	159.71 ± 9.57b	159.02 ± 15.08b	150.02 ± 18.37c
Succulence (mgH_2_O cm^−2^)	0.025 ± 0.00025a	0.027 ± 0.00052a	0.026 ± 0.00054a	0.026 ± 0.0009a	0.025 ± 0.0009a	0.025 ± 0.0009a

Results were reported in means (± SD). Means sharing dissimilar letters are significantly differed according to LSD test (*P* ≤ 0.05) for three flushes leaves aged (10–60 days) in Tables 8, 9 and 10.

### Chlorophyll and carotenoids contents of non-mined and mined leaves

**Figure 4:**
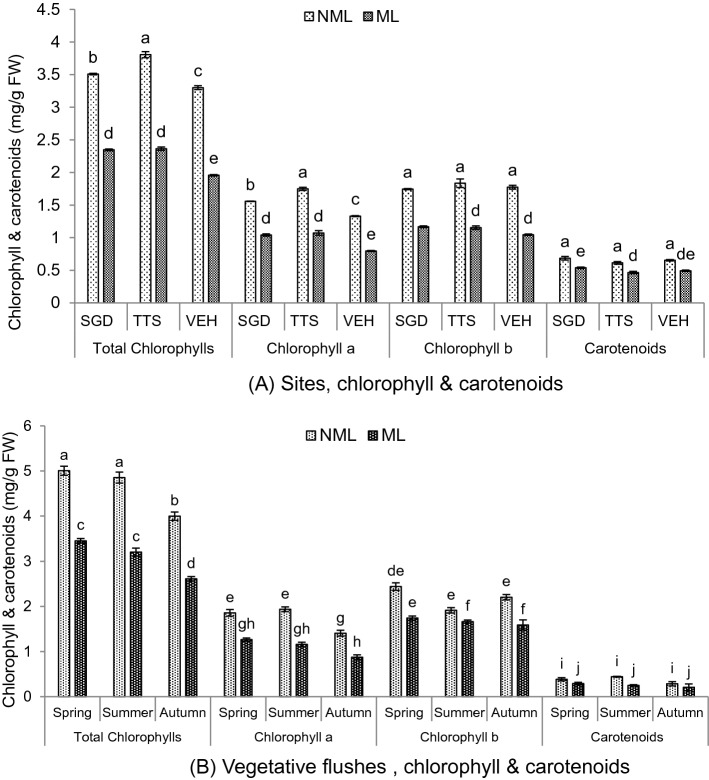
Chlorophylls and carotenoid contents of non-mined and mined leaves (**A**) different environmental conditions (**B**) three vegetative flushes. Bars sharing dissimilar letters are significantly differed according to LSD test (*P* ≤ 0.05). FW = fresh weight.

Chlorophyll and carotenoids content of non-mined and mined leaves are shown in Fig. 4. Total chlorophyll contents were estimated in non-mined leaves (3.51, 3.81 and 3.3 mg/g FW) and in mined leaves (2.35, 2.36 and 1.96 mg/g FW) in Sargodha, TTS and Vehari, respectively. Chlorophyll ***a*** was recorded in non-mined leaves (1.56, 1.75 and 1.33 mg/g FW) and in mined leaves (1.04, 1.07 and 0.8 mg/g FW) in Sargodha, TTS and Vehari, respectively. Chlorophyll ***b*** was calculated in non-mined leaves (1.75, 1.84 and 1.77 mg/g FW) and in mined leaves (1.17, 1.15 and 1.05 mg/g FW) in Sargodha, TTS and Vehari, respectively. Similar trends in carotenoid levels were seen in non-mined leaves (0.68, 0.61 and 0.65 mg/g FW) and in mined leaves (0.54, 0.47 and 0.49 mg/g FW) in Sargodha, TTS and Vehari, respectively. Leaves samples were collected from two months old summer flush, which showed a significant declining trend of both chlorophylls and carotenoids in the mined leaves at all three locations.

Total chlorophyll, chlorophyll ***a***, chlorophyll ***b*** and carotenoid contents were recorded as maximum in non-mined leaf (5.01, 1.86, 2.44 and 0.38 mg/g FW) and minimum in mined leaves (3.45, 1.26, 1.74 and 0.29 mg/g FW), respectively in spring flush. In summer flush, total chlorophyll, chlorophyll ***a***, chlorophyll ***b*** and carotenoid contents were maximum recorded in non-mined leaves (4.85, 1.94, 1.92 and 0.44 mg/g FW) and minimum in mined leaves (3.2, 1.16, 1.66 and 0.25 mg/g FW), respectively. Similarly, in autumn flush, total chlorophyll, chlorophyll ***a***, chlorophyll ***b*** and carotenoid contents were recorded as higher in non-mined leaves (3.99, 1.41, 2.2 and 0.28 mg/g FW) and lower in mined leaves (2.61, 0.87, 1.59 and 0.21 mg/g FW), respectively. Non-mined and mined leaves of spring and summer flushes were old while autumn flush leaves were young with low chlorophyll and carotenoid levels.

### Polyphenols and anti-oxidant activities of non-mined and mined leaves

Polyphenols and antioxidant activity of non-mined and mined leaves are presented in Table 11. Antioxidant activity {DPPH (1,1-diphenyle-1-2-picrylhydrazyle) inhibition} was recorded in non-mined leaves (27.66, 32.27 and 45.23 inhibition%) and in mined leaves (18.12, 18.07 and 32.40 inhibition%) respectively, in spring, summer and autumn flushes. Total phenolic contents were recorded in non-mined leaves (44.08, 68.08 and 41.73 mg of GAE/100 g) and in mined leaves (31.94, 56.01 and 28.17 mg of GAE/100gFW) in spring, summer and autumn flushes, respectively. Total flavonoid contents were recorded as maximum in non-mined leaves (16.97, 19.62 and 13.11 mg of quercetine equl/100 g FW) and minimum in mined leaves (10.74, 14.52 and 7.96 mg of quercetine equl/100 g FW) in spring, summer and autumn flushes, respectively. Similarly, total flavonols contents were found to be higher in non-mined (9.57, 10.71 and 8.24 mg of quercetine equl/100 g FW) and lower in mined leaves (5.85, 7.97 and 5.79 mg of quercetine equl/100 g FW) respectively in spring, summer and autumn flushes.

**Table 11:** Antioxidant activity and polyphenols in non-mined leaves (NML) and mined leaves (ML) of three vegetative flushes.

Polyphenols and antioxidant activities	Spring flush		Summer flush		Autumn flush	
	NML	ML	NML	ML	NML	ML
Anti-oxidant activities (DPPH inhibition %)	27.66 ± 0.64c	18.12 ± 1.86d	32.27 ± 0.32b	18.07 ± 1.79d	45.23 ± 0.39a	32.40 ± 1.48b
Total phenolic contents (mg of GAE/100 g FW)	44.08 ± 0.93c	31.94 ± 1.99d	68.08 ± 2.18a	56.01 ± 0.60b	41.73 ± 0.25c	28.17 ± 1.23d
Total flavonoid contents (mg of quercetine/100 g FW)	16.97 ± 0.13ab	10.74 ± 0.1c	19.62 ± 0.36a	14.51 ± 0.13b	13.11 ± 0.15b	7.96 ± 0.12d
Total flavonols contents (mg of quercetine/100 g FW)	9.57 ± 0.21ab	5.85 ± 0.22c	10.71 ± 0. 3a	7.99 ± 0. 25b	8.24 ± 0.25b	5.78 ± 0.2c

Results were reported in means (± SD). Means sharing dissimilar letters are significantly differed according to LSD test (*P* ≤ 0.05) for three flushes non-mined and mined leaves parameters.

### Physiological responses of non-mined and mined leaves

**Figure 5:**
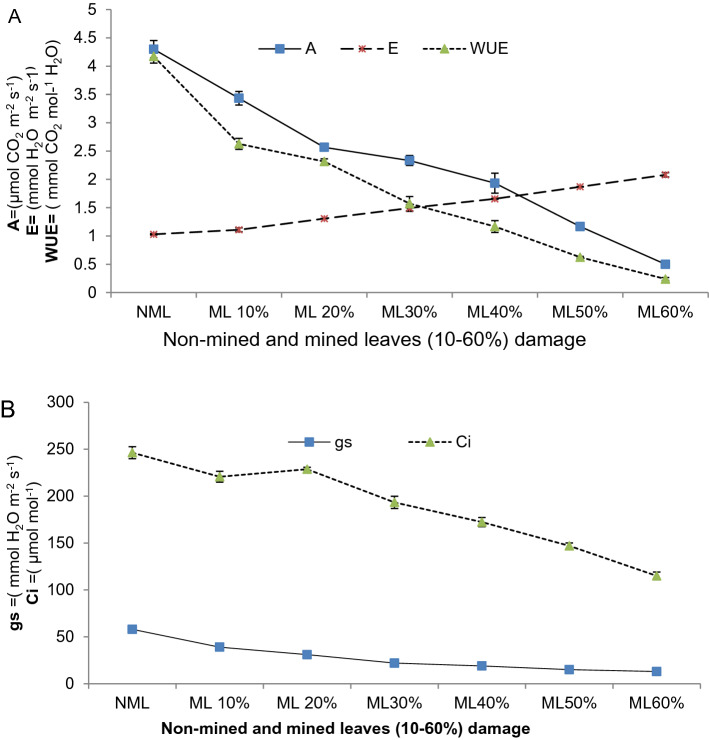
(**A**) Net assimilation rate (A), transpiration (E) & water use efficiency (WUE)and (**B**) stomatal conductance (gs) & sub-stomatal conductance (Ci) of non-mined and mined (different % damaged level).

Plant physiological activities of non-mined and mined leaves are presented in Table 12 and trend pattern in Fig. 5A,B. Physiological activity has shown a higher response in non-mined leaves than in mined leaves (10–60%) of damage levels. Maximum net assimilation rate (4.3 μmol CO_2_ m^−2^ s^−1^), stomatal conductance (58 mmol H_2_O m^−2^ s^−1^), sub-stomatal conductance (246.33 μmol mol^−1^) and water use efficiency (4.17 mmol CO2 mol^−1^ H_2_O) were recorded in non-mined leaves. In contrast to other physiological activity, there was a lower rate of transpiration in non-mined leaves (1.03 mmol H_2_O m^−2^ s^−1^) than in mined leaves (10–60%) of damage.

**Table 12:** Physiological responses of non-mined and mined leaves.

Physiological response	NML	Different level of mined leaves damage %					
		10% ML	20% ML	30%ML	40% ML	50% ML	60% ML
Net assimilation rate (μmol CO_2_ m^−2^ s^−1^)	4.3 ± 0.26a	3.43 ± 0.21b	2.57 ± 0.06c	2.33 ± 0.15 cd	1.93 ± 0.31d	1.17 ± 0.06e	0.5 ± 0.10f.
Stomatal conductance (mmol H_2_O m^−2^ s^−1^)	58 ± 1.73a	39 ± 1.15b	31 ± 2.0c	22 ± 1.15d	19 ± 1.0de	15 ± 1.0e	13 ± 1.0e
Transpiration (mmol H_2_O m^−2^ s^−1^)	1.03 ± 0.02e	1.11 ± 0.06d	1.31 ± 0.01c	1.49 ± 0.1bc	1.66 ± 0.02b	1.87 ± 0.01ab	2.08 ± 0.10a
Sub-stomatal conductance (μmol mol^−1^)	246.33 ± 11a	220.7 ± 10.2b	228.67 ± 3.51b	193.33 ± 11.24c	172.33 ± 8.5d	147 ± 5.29e	115 ± 7.0f.
Water use efficiency (mmol CO_2_ mol^−1^ H_2_O)	4.17 ± 0.20a	2.63 ± 0.17b	2.32 ± 0.08c	1.57 ± 0.22d	1.17 ± 0.18e	0.62 ± 0.03f.	0.24 ± 0.04 g

NML = Non-mined leaves and ML = Mined leaves (10%,20%,30%,40%,50% and 60% damaged leaves) Results were reported in means (± SD). Means sharing dissimilar letters are significantly differed according to LSD test (*P* ≤ 0.05) for non-mined and mined leaves different levels in Tables 8, 9 and 10.

### Photosynthetic activity of non-mined and mined leaf at different age

**Figure 6:**
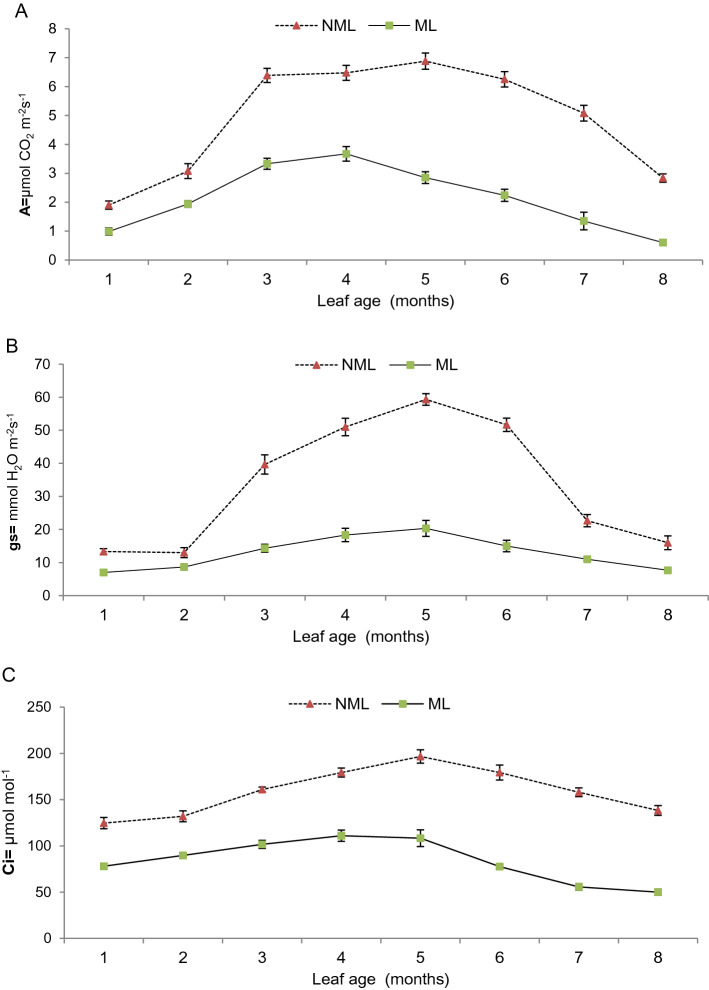
Photosynthetic activity of non-mined and mined leaves in leaf-age^1–8^ months (**A**) net assimilation rate (An) (**B**) stomatal conductance (gs) (**C**) sub-stomatal conductance/intercellular CO_2_ concentration (Ci).

Photosynthetic activities of non-mined and mined leaves are shown in Fig. 6A–C.

Maximum photosynthetic activities such as net assimilation rate, stomatal and sub-stomatal conductance were recorded in non-mined leaves and minimum in mined leaves. Increased trends in carbon assimilation, stomata and sub-stomata conductance/intercellular CO_2_ concentration were observed in old leaves (1–5 months) and then slowed down and began to decline in both non-mined and mined leaves. Maximum carbon assimilation (6.88 μmol CO_2_ m^−2^ s^−1^), stomatal conductance (59.33 mmol H_2_O m^−2^ s^−1^) and sub-stomatal conductance (196.67 μmol mol^−1^) were observed in non-mined leaves of 5 months compared to mined leaves.

### Leaf age of non-mined and mined leaves

The leaf-age of non-mined and mined leaves was shown in Table 13. In spring flush, non-mined leaf age was noted (292.67, 305, 320 days) and mined leaves (268.33, 276.33 and 296.67 days), respectively, at Sargodha, TTS and Vehari. Non-mined leaves of summer flush were recorded (250, 265 and 260 days) and mined (215, 240 and 235 days) in Sargodha, TTS and Vehari, respectively. Similarly, the autumn flush leaf-age of non-mined leaves were recorded (201.67, 195 and 185 days) and mined leaves (170, 160 and 151.67 days) in Sargodha, TTS and Vehari, respectively. In all three flushes, the leaf-age of the mined leaves was significantly reduced and a further reduction was observed in warm conditions.

**Table 13:** Leaf age of non-mined and mined leaves.

	Sargodha		T.T Singh		Vehari	
	NML (days)	ML (days)	NML (days)	ML (days)	NML (days)	ML (days)
Spring flush	292.67 ± 8.9ab	268.33 ± 6.03c	305 ± 5.89a	276.33 ± 6.48bc	320 ± 5.89a	296.67 ± 6.01b
Summer flush	250 ± 5.89d	215 ± 4.65f.	265 ± 5.87c	240 ± 4.89de	260 ± 4.35c	235 ± 5.35e
Autumn flush	201.67 ± 5.41 g	170 ± 4.82i	195 ± 5.89 g	160 ± 4.74ij	185 ± 5.96 h	151.67 ± 3.67j
LSD	13.14	12.12	9.98	13.73	9.99	9.11

Results were reported in means (± SD). Means sharing dissimilar letters are significantly differed according to LSD test (*P* ≤ 0.05) for both non-mined leaves (NML) and mined leaves (ML).

### Plant phenological growth trend in fluctuating weather conditions

**Figure 7:**
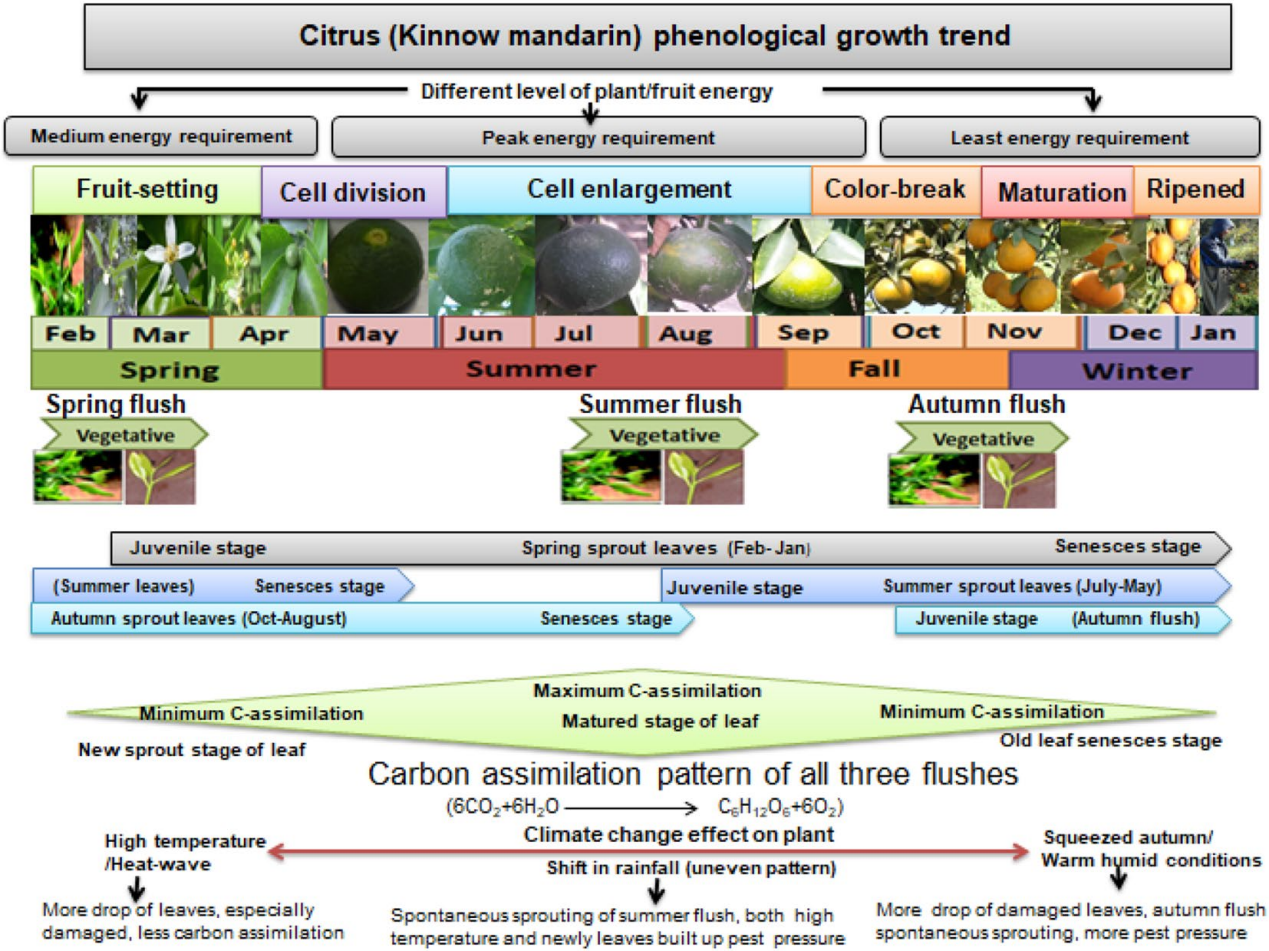
Phenological growth trend of citrus (Kinnow mandarin).

Phenological growth trend is given in Fig. 7 and based on field based study.

## Discussions

### Agrometeorological/Thermal indices

Temperature was recorded more at Vehari and average relatively humidity (RHa) at Sargodha^2^, henceforth more DDs, PTI and HTU were available at Vehari. Similarly, in Sargodha, DDs were counted less than in Vehari, but vice versa in the case of RHa; as a result, both districts had less HYTUs counts than TTS. Average relative humidity (RHa) was seen more at TTS than Vehari and mean daily temperatures were observed higher than Sargodha, henceforth more HYTUs were available at TTS on a monthly basis at different leafminer developmental stages. Bevington and Castle ^32^ reported that agrometeorological indices were fluctuated location-wise due to climatic factors variation in changing seasons. Similar results for additional growing degree days (GDDs) of crops have been reported in warm regions^33,34^. In the present work, mean daily temperature was recorded more at Vehari, followed by TTS and lower at Sargodha, therefore DDs were calculated by keeping different developmental stage threshold temperatures. The calculation of other agrometeorological indices were based on DDs and climate variables that also influenced the developmental stages of insect-pests^35^. Just as plant growth phases are directly linked to agrometrological indices, as in the case of citrus fruit growth phases^44^, the developmental stages of leafminer are also affected by climate variables^38^. DDs availability can determine the span of life cycle of leafminer and the number of generations all year round^42^. In addition, leafminer population pressure on citrus in a specific area is directly linked to the availability of agrometeorological indices^40^ and emerging flush^25^. More climate variations have been observed in citrus-growing three sites, and henceforth fluctuating agrometeorological indices have been computed^31^, while global warming has increased temperatures^20^ and biotic stress^19^. As a result, more DDs and other agrometeorological indices were available to leafminer at different developmental stages. More infestation was observed in TTS and Vehari in the present work to indicate that the leafminer egg to pupa stages took less time with more generations in a year. The developmental stages of leafminer (egg, larva and pupa) squeeze at high temperatures^25,43^. Nevertheless, different developmental process of leafminer ceases below threshold temperature^42^. Singh ^51^ also reports that leafminer has shown more growth and rapidly completed egg to pupa stage at high temperatures and prolongs developmental process in cool months while studying Kinnow and rough lemon plants. In current work, more DDs, PTI, HTU were computed in warm districts and summer months, so more pest infestation was observed in summer flush at TTS and Vehari. Pinto and Fucarino ^36^ and Santos et al. ^37^ report rapid developmental rates of different stages of leafminer (egg to pupa) in high photoperiod areas, while more HTU and PTI were available in warm regions and summer months in the current work to demonstrate that rising temperatures not only increased DDs but also increased agrometeorological indices in changing climate scenarios. Weather conditions decide on the available monthly basis agrometeorological indices^45^ to indicate the number of generations of leafminer throughout the year^41^ and the seasonal life cycle in a given area^39,40^. Fluctuating agrometeorological indices were computed in Kinnow growing three districts in climate change scenario, which has changed plant growth patterns in summer flush and also hastened the developmental process of leafminer as this work is justified by population model of insect pests based on meteorological factors and availability of resources^38^.

### Kinnow flushes and leaf miner damage

Newly sprouted leaves have thin epidermis and leafminer attacks are seen higher^60^. Similar results were observed in all three flushes in this study. In Kinnow mandarin, spring flush was counted 55–60%, followed by summer 25–30% and autumn 10–15%^50^ which justified current work on flushes. The highest oviposition rate of leafminer was recorded at 30 °C^25^ while similar temperatures were observed in the three districts during March. Relatively higher temperatures were recorded at Vehari and TTS during the last week of February to the end of March; more leafminer infestation was counted on newly sprouted spring flush leaves as this pest had overcome winter hibernation earlier. At high temperatures, more agrometeorological indices were available to leafminer and can quickly complete life cycle while squeezing the developmental stages (egg to pupa). Abo-Kaf et al. ^61^ report leafminer oviposition period 2.28 days at 30 °C which changes its duration in the changing temperature regime^25^. In current work, more agrometeorological indices were available to leafminer during the summer months, which produced overlapping generations. As a result, more larva mining was observed at TTS and Vehari, because in summer flush more growing degree days were accumulated to produce spontaneous vegetative growth. In autumn flush, less damage was seen due to low temperatures and no oviposition of the leafminer occurred at or below 15 °C^25^. Egg, larva and pupal developmental cycles were shortened with rising temperatures^25^. In warm conditions and summer months, more agrometeorological indices were available, while leafminer accelerated the life cycle by producing overlapping generations. As a result, more infestation was recorded in warm districts of Vehari and TTS than in Sargodha. Similarly, less agrometeorological indices were available in Sargodha in autumn and spring flushes, therefore less pest infestation was observed. In addition, heavy rainfall also slowed the growth of leafminer in the summer months, and more rainfall was reported in Sargodha, resulting in less infestation in the summer flush.

### Monthly sprouting and leaf miner larva entrance

Due to the winter hibernation of the leafminer, less infested leaves were reported on a monthly basis in February, but a sudden rise in temperature during March coincided with heavy spring flush, resulting in higher damage in all three districts as the optimal oviposition temperature available^25^. More agrometeorological indices were available in the warm districts of TTS and Vehari, with an increase in the pest population due to the rapid rate of developmental stages resulting in more infestations. High temperature in July to October optimized conditions for leafminer by recording more infestation in warm districts due to additional accretion of DDs, PTI and HTU. In the same way, the increase in the growing degree days (GDDs) in warm months led to spontaneous slow vegetative growth. As a result, newly sprouted leaves ensnared adult female for oviposition and hastily completed egg-hatching stage with more first instar larva population to feed tender young leaves. No vegetative growth occurred in the months of January, April, May, June and December, and henceforth no infestation was seen on mature leaves as the female preferred young leaves to lay eggs^62^. These findings are substantiated by the work of^63^ who reported that the peak mining period was February–March and July–October. Rainfall in Sargodha was higher than TTS and Vehari during the spring, summer and winter periods and has adverse effects on leafminer. Similar adverse effects of rainfall on the developmental stages of leafminers are observed during monsoon in Bangladesh^64^ justifying this study.

### Leaf size entrance by larva after egg-hatching

Mostly, the leafminer prefers young and tender leaves^8^ of a size (10–25 mm long) for oviposition^62^. The first instar larva hatched from the eggs immediately feeds on tender epidermis tissues and begins to mine zigzag in newly sprouted leaves^10^ while the first instar larva to the pupal stage continues to mine leaves to feed on spongy parenchymatous cells^60^. Maximum 60–63 percent of the larva entry was seen on a leaf size of up to 1 cm^2^, while 30 percent larva mining in the leaf size (1–2 cm^2^) was observed symmetrically in three districts. The larva making leaf size (2–3 cm^2^) mine was recorded to be the lowest in all districts (4–8%). Leaf size larger than 3 cm^2^ reached hardness for the entry of larvae or mine formation, suggesting that the small leaves (1–5 days) were more infested than the larger leaves. Leaf aged 11–15 days is resistant to entry/mining of larvae and leafminer population pressure on citrus is influenced by the availability of young leaves and weather factors^51^. Similar trends in newly emerging leaves have been observed in the present work, indicating that the larva preferred young, tender and emerging sprouts of all three flushes. Vercher et al. ^65^ also observed that the first instar larva feeds on young leaves (10–20 mm long) justifying extra attacks on small leaves in the present work.

### Leaf sclerophylly for leaf miner larva feeding after entrance

Female leafminer tends to lay eggs on emerging tender leaves^62^ and larvae enter epidermis soft tissues^10^ and make zigzags to feed on parenchymal spongy cells^60^. Among citrus leaf sclerophylly parameters, succulence determines leaf tenderness^50^. Citrus leafminer feeds on tender tissues of young leaves and prefers succulence value above 0.02 (mg H_2_O cm^−2^) which was recorded in spring and summer flushes up to 40 days of age. Succulence greater than 0.02 (mg H_2_O cm^−2^) was recorded in the autumn flush for up to 60 days. The leaf succulence in Kinnow mandarin allowed the larva to feed on the tissues for up to 40 days in spring and summer, while for 60 days in autumn. It suggested that the inner tolerance of the leaf against the entry of the larva was increased earlier in the spring and summer, and later in the autumn. The life cycle of leafminer depends on the availability of young leaves and external weather conditions^51^. In the spring and summer seasons, leafminer larva finished fast feeding while it extended feeding in the autumn due to tender tissue availability, in addition to being dependent on prevailing environmental conditions. In spring and summer flushes when more DDs were available, a rapid larva feeding was observed. While less DDs and other agrometeorological indices were calculated in late autumn and more leaf succulence was also recorded, which provided more space for larvae to feed until favorable external conditions were established for the next life cycle.

### Chlorophyll and carotenoids contents of non-mined and mined leaves

Chlorophylls and carotenoids pigments are essential in plants. Chlorophylls are actively involved in photosynthetic activities^66^, and channel solar radiant to assimilate atmospheric CO_2_ to organic carbon compounds^67^. However, carotenoids transmitted light for photosynthesis^68,69^ as well as protected leaves against harmful effects of solar radiation^70,71^ by stabilizing proteins in the photosystem^72^. In mined leaves, total chlorophyll, chlorophyll a and b, and carotenoid content were reduced due to feeding of mesophyll tissues and chloroplast depletion in leaves. Chen et al. ^15^ reported similar findings from mined leaves in a mangrove plant (*Avicennia marina*). Chlorophylls and carotenoids are directly involved in photosynthetic activities^73^ and ascertain leaf age on the tree while their reduction in mined leaves has slowed photosynthetic activity and reduced leaf age by inducing the cycle of leaf abscission^74^. In this study, chlorophylls and carotenoids were found to be lower in the mined leaves in all three districts and in the three vegetative flushes.

### Polyphenols and anti-oxidant activities of non-mined and mined leaves

Reactive oxygen and nitrogen species (ROS/RNS) are essential for chemical signaling, energy supply and defense mechanisms, but their overproduction under stress conditions or exposure to external oxidizing processes has caused failure of defense mechanisms and damage to key biochemicals such as DNA, protein, lipids^75^. Antioxidants can prevent oxidative damage^76^ and polyphenols have a positive antioxidant correlation^77^, while phenolic acids, flavonoids and flavonols are the main sources of citrus antioxidants^78^. Flavonoids have protein binding function while flavonoids bind proteins to cellular receptors and transporters^75^. Chlorophylls also work as an antioxidant compound in leaves^79^ which has been shown to be lower in mined leaves than non-mined leaves, and polyphenols such as total phenolic, flavonoid and flavonoid contents have a positive correlation with antioxidant activity, therefore less reported in leafminer infected leaves. Similarly, polyphenols have also been found to be lower in mined leaves due to loss of vital tissues and chloroplasts, indicating that mined leaves have less polyphenol and antioxidant activity than non-mined leaves.

### Physiological responses of non-mined and mined leaves

Non-mined leaf photosynthetic rates are higher than herbivores damaged leaves^17,80^, but leaf physiology is influenced by degree and damaged tissue types and C-compound relocation from primary to secondary metabolism^81^. A lower rate of net assimilation, stomatal conductance and water use efficiency were recorded in mined leaves than non-mined leaves. Similarly, leaf miner larvae feed parenchymatous tissues to varying degrees by affecting the physiological response of the leaf to the level of the mined/damaged leaves. As the damage to the leaves increased, photosynthetic activities have been more affected^82^. Similar trends have been recorded in mined leaves (10–60%) with a gradual decline in photosynthetic activity. Transpiration rate remains high in damaged leaves due to loss of epidermal and cuticle layers^83^ which also justified high transpiration in the Kinnow mandarin mined leaves in current work. Stomatal conductance recorded less in mined leaves that assimilated low carbon and also lowered photosynthetically water use efficiency^80^ while damage or loss of cuticle layer increased transpiration^83^. Similar findings in the current research have been recorded for damaged Kinnow mandarin leaves. Mined leaves have lost chloroplasts and degenerated thylakoids, which have reduced photosynthetic efficiency. Increased extent of mined leaves directly lowered net assimilation, stomatal and sub-stomatal conductance while accelerating transpiration rate. As a result, water use efficiency of leaf was declined. A similar trend was seen in this study of different levels of damage (10–60%). The present findings are consistent with the work of^18^ on *Pastinaca sativa* L. In contrast to non-mined leaves, decreases in stomatal activity in mined leaves^84^ have not lowered transpiration rates due to loss of cuticle layer^83^. As a rule, low carbon is assimilated and more water loss in transpiration, thus photosynthetically reducing the water efficiency of the leafminer infested leaves. Sub-stomatal conductance is actually inter-cellular CO_2_ concentration which has shown to be lowered in mined leaves due to damage of mesophyll and thylakoids tissues. As a result, low level of photosynthetic efficiency of the leaf was observed in damaged leaves of Kinnow mandarin. Similar findings are observed in the photosynthetic and gaseous exchange rates of citrus by recording a low physiological response of larval feeding leaves^80^.

### Photosynthetic activity of non-mined and mined leaf at different age

Photosynthetic activity of mined leaves remains lower^80,85^ due to feeding of parenchyma tissues^86^ that alter physiological performance of the leaf^85^. Significant reductions in net assimilation rate, stomatal and sub-stomatal conductance were observed in mined (1–8 months) old leaves compared to non-mined/intact leaves in the present work. Costa et al. ^16^ also reported low photosynthetic activity in leafminer damaged melon plants, which also confirmed low photosynthetic activity in mined leaves as recorded in this study. Dented chloroplasts in mined leaves reduce carbon assimilation^80^ and respiration rate^87^ due to low carbon accumulation or CO_2_ emissions in spongy parenchymal cells^88^ which reduce sub-stomatal conductance and ultimately delay the physiological response of damaged leaves^89^. In the same way, stomata below the mined area also responds to closure or opening but is impaired in function due to malfunctioning of the stomatal aperture that has decreased stomatal conductance^85^. Similar results were observed in recording of low net assimilation rate, stomatal and sub-stomatal conductance compared to non-mined leaves 1–8 months old. More reduction in photosynthetic activities was observed in mined leaves after the 5th month due to the prior start of senescence cycle. The present findings of low photosynthetic activity of the mined leaves are consistent with the work of^80,90^ on citrus^91^, on tomatoes and^92^ on Milkweed (*Asclepias syriaca*).

### Leaf age of non-mined and mined leaves

Leafminer damage young and tender leaves by mining on the epidermis tissues^10^. Leafminer feeds on parenchymatous cells^60^ and destroys mesophyll and thylakoid tissues as well as the cuticle layer^83^ and eventually deprives the leaves of vital components such as chlorophylls, carotenoids and polyphenols by reducing antioxidant activity^15^. In addition, mined leaves have low photosynthetic activity and incapacitated physiological response^85^. As a result, the leaf senescence cycle is accelerated as observed in current research. Mined leaves age was reported to be low than non-mined in all three Kinnow mandarin vegetative flushes. Mined leaves have a low physiological response^93^ and a weak defense mechanism against weather vagaries^94^. As a result, leafminer infested leaves reduced physiological efficiency and also reduced on-tree age by causing economic loss^95^ of less carbohydrate supply^80^ to the rest of the plant due to impaired xylem and phloem function^96,97^. In mined leaves, the abscission rate is more than intact leaves^74,98^, while infested leaves have active abscission^99^. The same trend of abscission was seen in this work of recording the earlier shedding of the mined leaves in all three flushes.

### Plant phenological growth trend in fluctuating weather conditions

Kinnow plant phenological growth trend in fluctuating weather conditions has favored more infestation of pests, especially leafminers. Floral and vegetative growth simultaneously begins in the spring season and maximum leaf growth and tender twigs are recorded about 60 per cent in spring flush^1^. Global warming has risen temperatures over the last century^20^ by inducing abiotic and biotic stress on crops^19^ and also on citrus^31^. In citrus growing areas, the autumn and spring seasons were squeezed^2^ while more agrometeorological indices were recorded under warm conditions and also during the summer months^44^. As a result, more growing degree days (GDDs) to plant and developmental degree days (DDs) to insect pests were available by altering the plant vegetative growth pattern and accelerating developmental stages of insect pests. Spring flush began maximum photosynthetic activity from fruit cell enlargement until the on-tree hanging fruits arrived at maturity. The second contribution of the net carbon assimilation is the summer flush, which provides energy to the hanging fruit in ripening stage as well as the newly sprouted floral and vegetative growth during the spring season. Autumn flush leaves assimilate less carbon at an early stage, which reached its peak during fruit-setting until the cessation of cell division. Leafminer damaged leaves of three vegetative flushes provide less carbohydrate due to low carbon assimilation in either hanging fruit or next-season crops. Fluctuating weather conditions have resulted in uneven growth patterns of leaves that favor overlapping leafminer generation to rapidly proliferate due to availability of more DDs. Fruit-set in the spring season, requiring maximum energy at cell division and cell enlargement phases, is likely to continue throughout the summer season. The autumn flush leaves assimilate maximum carbon by supplying energy to fruit during cell division, while the spring flush leaves are the main source of plant energy with an overwhelming source during the fruit cell enlargement phase and have also contribution in the maturing phase. Citrus leaf attained a maximum level of chlorophylls and carotenoids at the age of two and a half months, as the results begin with peak carbon assimilation, which declined at the senescence stage about 1 month before the leaves were shed. Mined leaves begin to shed ahead of time when other parts of the plant require more carbohydrate by causing direct economic loss of less energy supply to fruit^80^.

In climate change scenario, global warming caused extreme heat-wave at the start of summer season^2^ that has retarded net assimilation of carbon with earlier drop of summer flush leaves along with burning of tender leaves of spring flush. As a result, low carbon assimilation resulted in more fruit drops and warm-humid conditions in late summer, resulting in more carbon assimilation to spontaneously produce new sprouts with higher pest pressure, particularly leafminer. In the same way, both temperature and humidity increased during the autumn season, resulting in more carbon assimilation while inducing new sprouts to lure more pests until the beginning of winter. More leafminer pressure was observed in the current work at warm districts. Erratic weather behavior has adversely affected pest-damaging leaves in the climate change scenario; especially leafminer infested leaves. Climate change, on the one hand, has negatively affected the physiology of citrus plants; on the other hand, it has provided favorable conditions for the spread of pests, particularly leafminer.

## Conclusion

Leafminer has emerged as a serious citrus pest in a climate change scenario. More infestation was observed in the high-temperature TTS and Vehari districts during all three vegetative flushes, as more agrometeorological indices were available to accelerate the life cycle of the leafminer. More leafminer damage was recorded in the leaf size (0–1 cm^2^) while 12–15 days old leaves above 3 cm^2^ were tolerant. Spontaneous leaves grew more in TTS and Vehari between July and November than in Sargodha, with more leaves being mined. Mined leaves contained less chlorophylls, carotenoids and polyphenols with low antioxidant activity than non-mined leaves. The physiological response of the mined leaves remained low, affecting fruit yield and quality. These findings would be useful in the future to develop strategies by knowing the impact of leafminer on citrus in a changing climate scenario. Based on climate variables, the availability of agrometeorological indices will determine the size of the leafminer population and the number of generations over the year, as well as the growth pattern of the citrus plant vegetative flush.

## Dedication

This work is dedicated to Late Prof. Dr. Nadeem Akhtar Abbasi (1965–2021) of his sad demise on 29th March, 2021 due to COVID-19 Pandemic who supervised the research work of Rab Nawaz in PhD study.
